
JOURNAL INFORMATION
==============================
NLM Title Abbreviation: Inter Econ
Journal ID: Inter Econ
NLM Journal ID: 101086399

Inter Economics

ISSN: 0020-5346

ARTICLE INFORMATION
==============================
PMCID: PMC7385205
PMID: 32834093
DOI: 10.1007/s10272-020-0900-6
Article ID: 900
Article version: 1
Subjects: Editorial

Next Generation EU: A Large Common Response to the COVID-19 Crisis

Alcidi Cinzia cinzia.alcidi@ceps.eu

Gros Daniel danielg@ceps.be

grid.22793.3d 0000 0004 0609 4239 Centre for European Policy Studies (CEPS), 1 Place du Congrès, 1000 Brussels, Belgium
Electronic publication date: 2020 Jul 28
Print publication date: 2020
Volume: 55
Issue: 4
First page: 202
Last page: 203
Copyright: © The Author(s) 2020
License: Open Access This article is licensed under a Creative Commons Attribution 4.0 International License, which permits use, sharing, adaptation, distribution and reproduction in any medium or format, as long as you give appropriate credit to the original author(s) and the source, provide a link to the Creative Commons licence, and indicate if changes were made.
The images or other third party material in this article are included in the article’s Creative Commons licence, unless indicated otherwise in a credit line to the material. If material is not included in the article’s Creative Commons licence and your intended use is not permitted by statutory regulation or exceeds the permitted use, you will need to obtain permission directly from the copyright holder.
To view a copy of this licence, visit http://creativecommons.org/licenses/by/4.0/.
License URL: https://creativecommons.org/licenses/by/4.0/

issue-copyright-statement: © The Author(s) 2020

==============================

In July 2020, the European Council agreed upon the Next Generation EU (NGEU) plan to support member states hardest hit by the COVID-19 crisis with a €750 billion fund to be financed by new EU debt and to be added to the budget of the EU.

According to the Council conclusions, €672.5 billion (of the €750 billion total plan) will constitute the Recovery and Resilience Fund, which is to be disbursed as grants (€312.5 billion) and loans (€360 billion). The rest of the plan will be devoted to flexible cohesion policy grants to respond to the coronavirus crisis and support the green transition to a climate-neutral economy.

For a long time, a recurring criticism of the euro area has been the lack of a common central budget like that of mature federations. Has the COVID-19 crisis worked as a trigger for the creation of a new instrument that could be of strategic importance to the long-term stability of the euro area and more generally of the EU?

Two aspects are key to understanding whether this will be the case: the size and the allocation criteria, which define its purpose. Therefore, the first issue is whether this package is large enough to be considered a sensible common response to the current crisis. To answer, it is revealing to compare it with the national fiscal response of EU member states. According to the Spring Commission Forecast, the EU27 average (cyclically adjusted) deficit will be equal to about 4% of GDP in 2020. This is equivalent to about €550 billion, which is lower than the amount in the Recovery and Resilience Fund. If one adds the deficit forecasted for 2021, about €260 billion, given that fiscal measures will continue into next year, the total fiscal effort (the net of the automatic stabilisers) of member states will amount to about €800 billion. This is just above the Next Generation EU figure, suggesting that the EU contribution to the fiscal crisis measures is of a comparable size to those of member states. In addition, the SURE programme to support employment in member states with an envelope of €100 billion, is already in the implementation phase.

It should be recognised that national budgetary efforts also include large liquidity measures (tax deferrals, equity injections, early tax refunds, etc.) and credit guarantees which might become fiscal measures. But even on this account, the European contribution might be of a similar order of magnitude if one considers that the ECB has injected hundreds of billions of euros into the banking system to stabilise financial markets and ease credit conditions.

Moreover, per euro engaged, the economic impact of EU measures will be larger than that of national ones. In a number of EU member states, public debt is already at high levels. It is therefore possible that the expectation that deficits will lead to even higher debt levels, and thus increase taxes, will make consumers more prudent, leading them to save a large part of the income they are receiving now. This effect, the so-called Ricardian equivalence, should be less relevant for EU-financed expenditure, which does not lead to an outright increase in (national) public debt. So from a size perspective, one can say that the Next Generation EU is a suitable instrument for crisis response.

The main purpose of the Next Generation EU is multifaceted and defined as “[t]o ensure the recovery is sustainable, even, inclusive and fair for all Member States”. According to the Council conclusions, €47.5 billion will be allocated based on the severity of the socio-economic impacts of the crisis under the REACT-EU programme. The resources, presented as a top-up to current cohesion policy programmes but without co-funding, are expected to be disbursed between now and 2022 based on GDP losses as well as the level of youth unemployment and the relative prosperity of member states. REACT-EU, while limited in size, is a true fiscal measure to mitigate the impact of the COVID-19 shock.

By contrast, the Recovery and Resilience Facility, the largest part of Next Generation EU, offers financial support to member states for investments and reforms in relation to the green and digital transitions and to augment the resilience of national economies. This means that the use of the funds is only partially linked to the impact of the crisis, and rather attached to the achievement of wider objectives.

Seen from this perspective, the bulk of Next Generation EU is not expected to have a shock-absorbing function, which is typical of central fiscal capacity in federations. Its purpose resembles the traditional EU budget where common financial resources are pre-allocated at the beginning of programming period, should be used to achieve EU objectives and are conditional upon strengthening national economies.

One drawback of such duality of the NGEU between the overarching idea of a plan to respond to the COVID-19 crisis and the actual approach to link funds to the EU objectives of resilience, sustainability and fairness is that it made it more complex to define the allocation of funding.

In practice, it will be more difficult to make sure *ex ante* that the countries hardest hit will also receive more resources. In the end, the allocation formula will be based on the Commission forecasts and linked to past unemployment rates, as well as population, inverse GDP per capita and youth unemployment, similar to the EU criteria for the allocation of cohesion funds. For 2023, unemployment criterion is replaced by the loss in real GDP observed in previous years. Introducing the idea that the NGEU funds should be pre-allocated weakened the idea that it could serve as a shock absorber, and emerged as a double-edged sword: a way to strike a compromise but at risk of poisoning the negotiations.

With pre-allocation, member states feel justified to follow the principle of 'juste retour', trying to maximise how much they can obtain in terms of grants or loans, reducing the share of grants and adding conditionality. Shock absorption does not entail any form of conditionality or earmarking system. Both are determined by the shock itself. It was expected even before the Council that some member states would not be ready for such a step.

Indeed, negotiations were long and tough. Strong political divisions within the Council – between the "frugal four" countries (Austria, Denmark, the Netherlands and Sweden) and those hardest hit by the crisis (Italy, France and Spain) – emerged. Conditionality to ensure that the money is not regarded as wasted gained currency among the frugal countries who pledged fewer grants and more loans, compared to the original Commission proposal, but also a more complex procedure[1] to approve the allocation of funds *ex ante* and for the EU Council to maintain the power over resources even in the implementation phase.

This approach makes it difficult to consider NGEU as a genuine fiscal capacity to mitigate the impact of the COVID-19 shock. In the end, however, an agreement was reached that is a combination of shock absorption and an extension of the EU budget. It reflects a politically acceptable compromise to put in place the first large EU common crisis response.

1 This would include submitting detailed national "recovery and resilience plans" on the basis of country-specific recommendations made by the Commission in the context of the European Semester.

Cinzia Alcidi, Centre for European Policy Studies, Brussels, Belgium.

Daniel Gros, Centre for European Policy Studies, Brussels, Belgium.

